# Non-Hodgkin's Lymphoma Within a Breast Abscess in a Male Patient: A Presentation and Literature Review of a Rare Case

**DOI:** 10.7759/cureus.67601

**Published:** 2024-08-23

**Authors:** Farah F Kazi, Shane Zahra Batool, Ahmed Kazi, Yasir Bashir

**Affiliations:** 1 General Surgery, Midland Regional Hospital Tullamore, Tullamore, IRL; 2 Surgery, Tullamore Hospital, Tullamore, IRL; 3 Surgery, Royal College of Surgeons in Ireland, Dublin, IRL

**Keywords:** non-hodgkin's lymphoma, breast disease, rare cancers, rare breast mass, general surgery and breast cancer

## Abstract

Breast abscesses are a common cause of presentation to the hospital. These should be treated with caution due to the possibility of rare pathology.

We present a rare case of a 59-year-old diabetic gentleman who presented to the emergency department with a two-day history of a large right-sided breast swelling along with an area of induration, consistent with an abscess, extending to the right axillary region. Initial laboratory findings revealed elevated inflammatory markers. He was admitted for intravenous antibiotics.

A computed tomography (CT) of the thorax performed on admission showed an ill-defined collection in the subcutaneous tissue of the right breast and axilla and an irregular right-sided peribronchial nodule with multiple enlarged pathological lymph nodes.

This patient's case was discussed with tertiary specialist breast services and local respiratory teams. He underwent an ultrasound-guided right axillary lymph node biopsy. The histopathology of this revealed a high-grade malignant non-Hodgkin's lymphoma of the diffuse large B-cell (DLBCL) type. He was referred for a positron emission tomography (PET) scan and hematological oncology services for further treatment in the form of chemotherapy.

This case presentation brings forward the importance of considering rare diagnoses and unusual histopathology when assessing a male breast lesion.

## Introduction

Breast abscesses are a common cause of presentation to the breast outpatient clinic and the emergency department. While these occur predominantly in lactating women, breast abscesses can also occur in males, and treating surgeons should keep a high index of suspicion for abnormal or atypical pathology.

Breast abscesses in lactating women are typically bacterial in etiology with *Staphylococcus aureus* as the most common causative organism [1]. If left untreated, these can expand to form fistulous tracts [1]. The histopathology of an abscess wall or membrane often demonstrates a dense inflammatory infiltrate with predominant neutrophils [1].

Non-Hodgkin's lymphoma (NHL) is a diverse group of lymphoid malignancies that can affect various parts of the body, including extranodal sites. It is most commonly associated with the growth of malignant lymphocytes.

Breast involvement in NHL is rare, with an incidence of <1% [2], and the simultaneous presence of a breast abscess can present a unique diagnostic and therapeutic challenge for the general surgeon.

This overview will discuss the diagnosis of NHL in the context of a breast abscess, including clinical presentation, diagnosis, and management.

## Case presentation

We report the case of a 59-year-old gentleman presenting to the emergency department with a two-day history of a large right lateral chest wall swelling and pain.

On examination, there was a large area of diffuse erythema in the right anterolateral and lateral aspect of the chest, with a focus of fluctuance extending below the right axilla, consistent with a right-sided breast abscess with surrounding induration and cellulitis. There was no evidence of skin retraction of the breast, peau d'orange appearance, or nipple retraction or discharge. The patient denied a history of trauma, previous infection, or surgery to this region. 

Laboratory findings revealed an elevated white cell count and C-reactive protein (CRP) as shown in Table 1.

**Table 1:** Inflammatory markers on presentation

Blood test investigation	Patient's value	Normal range	Unit
White blood cells	17.2×10^9^/L	4-11×10^9^/L	×10^9^/L
C-reactive protein	231 mg/l	0-5 mg/l	mg/l

This gentleman was also noted to have a background history of prostate cancer, treated with previous prostatectomy combined with radiotherapy, type II diabetes mellitus, and hypertension along with being a smoker. He had no significant family history of any breast, visceral, or hematological malignancy.

**Figure 1:**
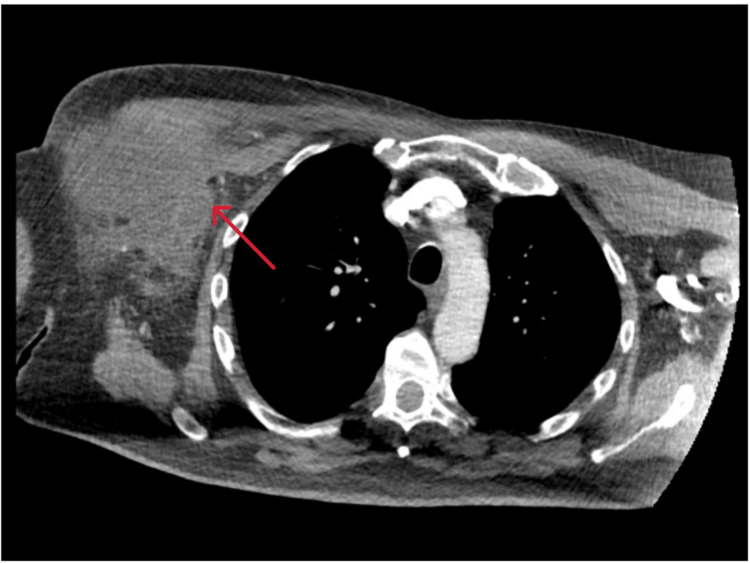
Computed tomography image showing a collection in the subcutaneous tissue of the right axilla and right breast

On presentation, he underwent a computed tomography (CT) which suggested a thick-walled, irregularly shaped, and ill-defined collection with a maximum diameter of about 12×6×8 cm in the subcutaneous tissue of the right axilla and right breast. Furthermore, there were associated multiple enlarged lymph nodes in the right breast, axilla, and right supraclavicular region (Figure 1).

**Figure 2:**
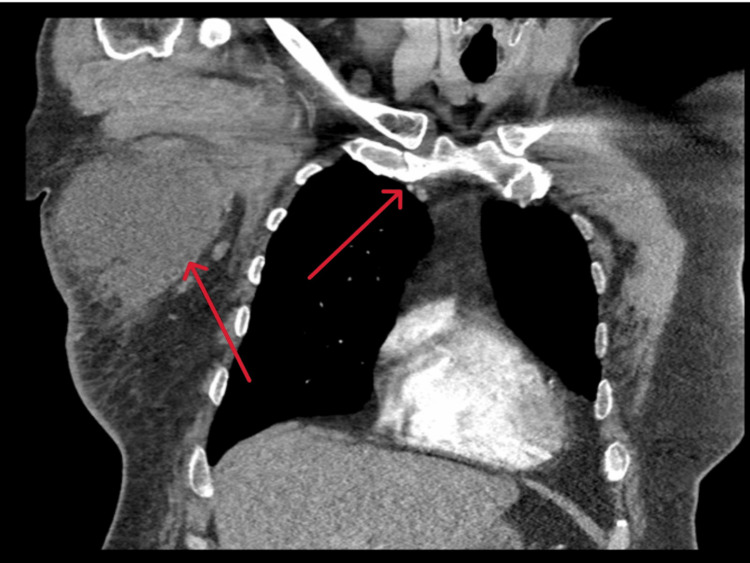
Computed tomography image showing an irregularly shaped peribronchial nodule in the right upper lobe along with the collection in the right axilla and breast

In the CT imaging, a suspicious irregularly shaped peribronchial nodule in the right upper lobe was also seen, measuring 25 mm, along with a few non-specific, partially calcified pulmonary nodules in the right middle and lower lobes (Figure 2).

Intravenous broad-spectrum antibiotics, fluids, and analgesia were commenced, and the area of erythema was demarcated to assess for interval progression or resolution.

An opinion was sought from the respiratory services for the peribronchial nodule in the right upper lobe and additionally from the breast unit in a tertiary center. Collectively, it was advised to obtain an ultrasound-guided biopsy of the lymph nodes to facilitate tissue analysis.

An ultrasound-guided biopsy of the axillary lymph node was performed. The final histopathological examination revealed a high-grade malignant non-Hodgkin's diffuse large B-cell lymphoma (DLBCL). The cores of fibroconnective tissue infiltrated atypical lymphoid infiltrate, positive for CLA, CD20, BCI2, CD10, and BCI6 with high MIBI (proliferation marker) >80%. No staining was seen with S100, CK20, CK7, CD23, and AE1/AE3. A positron emission tomography (PET) scan showed stage 4S high-grade lymphoma with extranodal involvement in the liver, multiple bone sites, and right chest wall.

Following the diagnosis, after the review by the hematology services, treatment was commenced, consisting of six biweekly cycles of the rituximab, cyclophosphamide, doxorubicin hydrochloride, vincristine sulfate (Oncovin), and prednisolone (RCHOP) regime.

This case underwent discussion at the lymphoma multidisciplinary team meeting and was planned for long-term intravenous methotrexate. Interval follow-up PET CT suggested the complete resolution of the lymphoma, with a persistent peribronchial lesion. This was assessed via bronchoscopy, and biopsies were taken.

The patient has been reviewed regularly, most recently seen in the clinic eight months following the initial presentation to the hospital, and is doing well.

## Discussion

Breast lymphomas (BL) are a rare entity of extranodal lymphomas [2]. These can form as part of primary breast tumors or as a result of a secondary metastatic process [2]. BL can present variably, with either local symptoms, similar to those described in this case report, or more widespread systemic symptoms [2]. Patients may also present with the characteristic B-symptoms of lymphomas, including night sweats, fevers, and/or weight loss [3].

In the literature, Wiseman et al. describe the criteria for the diagnosis of BL. This includes adequate pathologic evaluation, mammary tissue in close association with lymphomatous infiltrate, no evidence of disseminated lymphoma, and no prior diagnosis of lymphoma [4]. As there is a comparatively lower amount of lymphoid tissue in the breast, BL is a rarely diagnosed pathology [5].

The most commonly used system for staging NHL is the Ann Arbor system. This uses a combination of radiological CT findings and clinicopathologic features. Stage 1 is described as the involvement of one lymph node group, stage 2 as the involvement of two or more nodal groups on the same side of the diaphragm, stage 3 as involvement on both sides of the diaphragm, and stage 4 as disseminated organ involvement [6].

There are various subtypes of NHL, namely, DLBCL, follicular lymphoma, mantle cell lymphoma, and Burkitt lymphoma (BkL) [7].

Primary BLs are largely of B-cell origin, and over 50% are diagnosed with DLBCL, followed by follicular B-cell lymphoma, extranodal marginal lymphoma, and BkL [8]. These variants have different epidemiologies, diagnoses, treatments, and prognoses; therefore, accurate histopathological analysis is essential in aiding in prompt and correct treatment. The use of adequate immunohistochemical staining is highly important in differentiating between subtypes [9].

Current literature suggests that unlike neoplasms originating from the breast tissue, mastectomy or any form of surgical intervention is associated with poorer prognosis and the rate of recurrence is higher [9].

BLs can present in the form of breast pain, swelling, solitary lump, skin changes, and nipple discharge and retraction, similar to a breast carcinoma [9]. This is particularly of importance as it is challenging to differentiate between the two entities. Along with variable clinical presentations, BLs vary in terms of histology and immunohistochemical features.

## Conclusions

BLs are a rare subgroup of primary and secondary breast malignancies. This case reports a diagnosis of NHL within a breast abscess. Prompt recognition and adequate immunohistochemical staining by an experienced histopathologist is necessary to determine the specific subtype of NHL present, as each subtype has a different treatment regimen and prognosis. Early involvement of a breast surgeon and other relevant specialists is essential to ensure the best possible outcome for patients. Our case also emphasizes on the importance of a multidisciplinary approach to ensure a positive outcome for the patient. A collective input enables an optimal streamlined management and allows for an appropriate follow-up in clinics.
